# Dichotomous roles of co-stimulatory molecules in diabetes mellitus

**DOI:** 10.18632/oncotarget.23102

**Published:** 2017-12-07

**Authors:** Ji-Xin Zhong, Jie Chen, Xiaoquan Rao, Lihua Duan

**Affiliations:** ^1^ Department of Endocrinology, Central Hospital of Wuhan, Wuhan, Hubei, China 430061; ^2^ Cardiovascular Research Institute, School of Medicine, Case Western Reserve University, Cleveland, Ohio, USA 44106; ^3^ Basic Medical Department of Medical College, Xiamen University, Xiamen, China 361102; ^4^ Department of Rheumatology and Clinical Immunology, The First Affiliated Hospital of Xiamen University, Xiamen, Fujian, China 361003

**Keywords:** co-stimulatory molecule, diabetes mellitus, dendritic cells, macrophage, CD28

## Abstract

Numerous studies have established the importance of immune dysfunction in the development of diabetes mellitus, including typ1 and typ2 diabetes, and it is worth noting that T cell activation acts a key role in the pathogenesis of loss of β cell mass, adipose inflammation and insulin resistance. Regarding as an important checkpoint in the process of T cell activation, co-stimulatory molecules interaction between antigen present cells and T cells have been identified the critical role in the development of diabetes mellitus. Thus, blockage of co-stimulatory dyads interaction between antigen present cells and T cells was supposed to a potential of therapeutic strategies. However, studies also showed that inhibition or deletion of some co-stimulatory molecules do not always reduce the development of diabetes, and even exacerbate the disease activity. Here, in this context, we highlight the dichotomous role of co-stimulatory molecules interaction in the pathogenesis of diabetes.

## INTRODUCTION

Diabetes mellitus is characterized by chronic hyperglycemia, which is resulted from the loss of β cell mass or loss of insulin sensitivity [1–3]. Long-term improper control of blood glucose homeostasis predisposes patients to the development of diverse complications such as diabetic retinopathy [4], nephropathy [5], neuropathy [6], foot ulcers [7], and cardiovascular diseases [8]. Now it is well-accepted that abnormal immune response including both innate and adaptive immunity plays a critical role in the development of β cell destruction and insulin resistance, although the underlying mechanisms remains elusive [9–11]. The co-stimulatory molecules are important regulators of immune activation via providing second signal for T cells activation [12]. Therefore, most studies have shown that interaction of co-stimulatory molecules exhibits a pathogenic role in autoimmune diseases [13–15]. Remarkably, B7/CD28 interaction is the vitally important second signal [16–18]. However, increasing evidence has suggested that some co-stimulatory signaling pathways may have a protective role in diabetes pathogenesis. Here, we review dichotomous role of these co-stimulatory molecules serve complicated roles in diabetes mellitus, especial B7/CD28.

### Co-stimulatory molecules and diabetes

When the body encounters foreign antigens or endogenous danger signals, immune response usually initiates. The innate immune cells, especially the professional antigen present cells (APCs), play a crucial role in uptaking and processing pathogenic substance present the antigen to T cells through MHC molecules [12, 18], which results in cellular and humoral immunity. In addition to presenting antigens to T cells, APCs also express co-stimulatory molecules which are regulated by the exogenous pathogens or endogenous alarmins [such as high mobility group protein 1 (HMGB1), heat shock protein 70 (HSP70) as well as interleukin-33 (IL-33)], providing the second signal for T cell activation [19]. The second signal is initiated by the interaction between co-stimulatory molecules expressed on the APCs and their corresponding ligands on the surface of T and B cells [20]. Like the neural synapse, the co-stimulatory dyads form an immunological synapse which is very important for the T or B cells activation [21]. In the absence of the second signal, the interaction of antigen-MHC complex with TCR or BCR, also known as the first signal, is insufficient to activate the T cells or B cells, leading to T cell and B cell anergy or apoptosis [20]. A number of co-stimulatory molecules have been identified, among which CD28/B7, CD40L/CD40, PD-1/PD-L1 and ICOS/ICOSL are the best-characterized co-stimulatory dyads involved in the immune synapse and immune cells activation.

Type 1 diabetes mellitus (T1DM) is the consequence of the autoimmune mediating pancreatic insulin-producing β cells damage and loss [3]. Inflammatory autoreactive T cells, escaping from central and peripheral tolerance recognize pancreatic islet antigens, can be activated in the pancreatic lymph nodes. Activated T cells then migrate to local pancreatic islet and induce an inflammatory micro-environment, recruiting macrophage and neutrophil infiltration and leading to pancreas islet damage and injury [22]. Of note, obesity-associated inflammation is widely believed to play a key pathogenic role in the development of obesity-induced insulin resistance and type 2 diabetes mellitus (T2DM). Innate immune activation typified by infiltrating macrophages is deemed to represent important mediators of obesity-related complications [11, 23, 24]. Keeping with previous reports, our recent work also demonstrated that a critical role acted by adipose macrophages in T cells immune responses during this process [25].

The co-stimulatory molecules mediating the interaction between T cells and APCs have been linked to the development of abnormal immune response [20]. Therefore, inhibition of co-stimulatory molecules interaction has been suggested to modulate T cell activation. Lots of studies have indicated a protective role of co-stimulatory inhibition in the development of many disease, such as experimental allergic encephalomyelitis (EAE) [26–29], allograft transplantation [30–32], arthritis [33, 34], and hypertension [35, 36]. Furthermore, the abnormal immune response induced by co-stimulatory molecules also result in β cell loss and insulin resistance in T1DM and T2DM [9, 22, 37]. Thus, dampening inflammation induced by autoimmune response become a potential therapeutic method in diabetes. However, increasing evidences suggest a protective role by some co-stimulatory molecules dyads in diabetes pathogenesis. Below we review the complicated roles of co-stimulatory molecules dyads in the development of diabetes.

### Role of B7/CD28 in diabetes

Two signals are required for full activation of naive CD4+ T lymphocytes as described [20]. T cell will be anergy or undergo apoptosis in the absence of second signal [38]. Therefore, co-stimulation inhibition shows a great therapeutic potential in immune-mediated diseases. B7 molecules, including B7-1 (CD80) and B7-2 (CD86), are the best-characterized co-stimulatory molecules and mainly expressed on APCs such as dendritic cells (DCs), B cells, and macrophages [17]. Nevertheless, Study have shown that B7-2 might be more important in the initiation of immune responses, as its expression is rapidly up-regulated when APCs encounter endogenous damage alarmin or foreign bodies, yet B7-1 is up-regulated in the later phase during the immune response [39, 40]. Through binding its specific receptor CD28, B7 activates protein kinase Cθ (PKCθ) and RAS guanyl nucleotide-releasing protein (RASGRP) [41, 42], which promotes T cell activation, proliferation, and anti-apoptosis. Of note, the co-inhibitory receptor CTLA-4 also shares the ligands (B7-1 and B7-2) with CD28 [40]. Generally, CTLA-4 is highly expressed in activated T cells, which is the self-control for preventing excessive immune response by binding to B7-1 or B7-2 [29, 43]. This co-inhibitory receptor CTLA-4 attracts increasing intention, because of its potential in immune regulation and higher binding affinity compared with CD28. CTLA-4 Ig has been used in the treatment of autoimmune disease [29, 33] and transplant rejection [44, 45]. Overall, the opposing roles of CD28 and CTLA4 are considered a prototypical immune checkpoint for the immune response through competing pro- and anti-inflammatory effects.

Interestingly, studies have demonstrated that basal B7-1 and B7-2 expression is also necessary to prevent autoimmunity by sustaining regulatory T (Treg) cell populations [46–49]. In our previous investigation, we also elucidated a homeostatic role of B7-mediated co-stimulation in diet-induced obesity using CD80/CD86 double knockout (B7 KO) mice and investigated the relevance of this process in humans with obesity and IR [50]. Our results suggested an essential role for B7 in maintaining Tregs and adipose homeostasis and may have important implications in immunotherapies targeting co-stimulation in type 2 diabetes.

As is well-known, interaction between B7 and CD28 promotes inflammation, while there is difference between the function of B7-1 and B7-2. In the development of type 1 diabetes in the NOD mouse, a distinct regulation of B7-1 and B7-2 were observed. At the onset of insulitis, mice treated with CTLA4 Ig or a blocking B7-2 antibody did not develop diabetes. However, there is no significant effect when CTLA4 Ig or a blocking B7-2 antibody administered late. Consistently, a delayed development of diabetes was seen in B7-2 knockout NOD mice, where islet-reactive CD4 T cells were defective. In contrast to the effect of B7-2 inhibition, B7-1 neutralization or gene deficiency causes exacerbation of disease, the lack of B7-1 significantly accelerated the development of disease accompanied by enhanced expansion, survival, and effector function of islet specific T cells in periphery [51, 52]. Furthermore, B7-1 deficiency mice showed a significant reduction in immunosuppressive Tregs cells [52]. These results suggest that B7 may play complicated role in the development of autoimmunity. Likely, in our previous study, expression of B7-1 and B7-2 was negatively correlated with the degree of IR and adipose tissue macrophage infiltration in both humans and mice. Furthermore, instead of promoting inflammation, ablation of CD80/CD86 by double gene knockout defects Tregs development and proliferation in mice, and exhibits enhanced adipose macrophage inflammation and IR under high-fat diet feed. Conversely, adoptive transfer of Tregs reversed IR and adipose inflammation in B7 KO mice [50]. Taken together, above studies of B7/CD28 co-stimulatory molecules show a complicated role in development of immune-mediated disease, including diabetes.

### Beneficial roles of B7/CD28 in diabetes

Although B7/CD28 co-stimulation participates in the induction and progression of autoimmune diseases, it has also been demonstrated that B7/CD28 co-stimulatory molecules interaction is substantial for Tregs development and proliferation. To examine the role of B7/CD28 in the development of EAE, CTLA-4 Ig was administrated to the mice. Unexpectedly, B7 blockade with CTL1-4 Ig exacerbated disease signs and exhibited more severe CNS inflammation and demyelination, which was associated with the increased inflammatory cytokines IL-17 and IFN-γ [29]. Similarly, in our previous study, CD80/CD86 was found to be essential for Tregs development and proliferation in obese mice and human, instead of promoting inflammation [50]. Furthermore, a subpopulation of CD4+ T subsets, characterized by low CD28 expression, is resistant to apoptotic signals and lives longer *in vivo* [53, 54]. The CD4 + CD28- T cells shows an atherogenic and plaque-destabilizing property [55–59]. It is well known that the diabetes patients are at high risks of atherosclerosis. Therefore, these T cell subpopulations were investigated in diabetes patients. When compared with non-diabetic individuals, T2DM patients with proliferative diabetic retinopathy showed a higher percentage of CD4 + CD28- population [60]. A study showed that CD4 + CD28- T cells potentially drive the severity of the disease through producing IL-17, and IL-17 expression of CD4 + CD28- T cells was regulated by NKG2D. In addition, when compared to non-diabetic individuals, CD4 + CD28- NKG2D + T cells subpopulation is increased in T2DM patients [61]. Shi B et. al showed that advanced glycation end products (AGEs) effectively enhanced these subset T cells proliferation in patients with T2DM, and the higher level of CD4 + CD28- T cells is closely associated with the status of macrovascular atherosclerosis in patients with T2DM [62]. Similarly, by means of ultrasound image to analyze the atherosclerotic plaque in the common carotid artery (CCA), CD4 + CD28- lymphocytes reveals a positive correlation with the number of atherosclerotic plaques within the CCA. In a clinical follow-up observation, CD4 + CD28- T cells are correlated with the occurrence of a first cardiovascular event and with a worse outcome after an ACS in DM patients [63]. These data revealed that the expression of CD28 molecules on CD4 + cell is vital for immune homeostasis in T2DM.

Furthermore, lack of B7/CD28 interaction also results in a limited numbers of regulatory T cells and aggressive disease progression in the T1DM NOD mice [64]. Treg cells were markedly decrease in the B7-1/B7-2-deficient and CD28-deficient NOD mice [49]. Additionally, the percentage of CD4 + CD28 + T cells and IL-2 production were also decreased along with aging, which resulted in impired Tregs function in NOD mice [65]. Recently, multipotent stem cells received huge attention in the treatment of many diseases due to its immunoregulatory and tissue repair functions [66–70]. In a clinical trial, the C-peptide levels, median glycated hemoglobin A1C (HbA1C) values, and the median daily dose of insulin were markedly improved in T1DM patients treated with cord blood-derived multipotent stem cells. Study also showed that the improvement was associated with increased expression of CD28, ICOS and the number of Tregs [71]. This study sustains the concept that CD28 plays an immunoregulatory function. Keeping with above reports, B7-1 gene knockout NOD mice showed a diminished amount and expansion in Tregs, accompanied by increased survival and amplification of auto reactive T cells [52]. Bour-Jordan H et.al further demonstrated that B7-1 overexpression on B cells completely protected NOD mice from developing diabetes [72]. These studies suggest a protective role of B7/CD28 in the development of diabetes.

### The pro-inflammation of B7/CD28 in diabetes

As is well-known, the interaction between B7 and CD28 is critical for the second signal of T cell activation and promotes inflammation [18]. In a case report, the patient with T2DM showed a dramatic improvement in insulin resistance when blockage CD28 activity by CTLA4-Ig infusion [73]. Furthermore, high glucose conditions promoted podocytes to express B7-1 both *in vitro* and *in vivo*. Treatment with CTLA4-Ig inhibited the apoptosis of podocytes, leading to an improvement of urinary albumin excretion and kidney pathology in these animals. Besides, the B7-1 expression is also up-regulated in podocytes from kidney biopsy specimens of T2DM patients [74]. Moreover, the expression of B7-2 has also been shown to increase in gestational diabetes mellitus (GDM) patients [75, 76]. Although not statistically significant (probably due to the small sample size), Schliefsteiner et al. reported that there was an increase of B7-2 in parallel with proinflammatory cytokines IL-1β and IL-6 in patients with GDM [76]. The expression of CD28, the binding partner of B7, was also increased in the peripheral T cells from patients with GDM [77].

A single-nucleotide polymorphisms (SNPs) analysis demonstrated that CD28 might contribute to the risk of T1DM [78]. In addition, a recent study showed that mice deficient for CTLA-4 or treated with anti-CTLA-4 antibody exhibited spontaneous follicular T cells (Tfh) differentiation by enhancing the strength of CD28 ligation with B7-1 and B7-2 [79]. IL-21, a critical cytokine in autoimmunity, can promote autoimmune response through up-regulating B7-2 on B cells [80]. These studies showed a great potential of B7/CD28 in the treatment of autoimmune diseases. Indeed, a marked reduction of spontaneously activated CD4 T cells and islet-specific CD4 T cell expansion and enhanced CD4 T cell death were observed in B7-2 knockout NOD mice. Interestingly, a significant reduction of Treg was not seen in the peripheral compartments of B7-2 KO mice [81]. Contrary to the inflammatory characteristic of CD4 + CD28- subset T cell, a CD8 + subpopulation with lower expression of CD28 exhibits an anti-inflammatory function [82]. In the peripheral blood mononuclear cells from juveniles with T1DM, CD8 + CD28- T cell subset was significantly reduced and correlated with disease duration. Moreover, CD8 + CD28- subpopulation was also significantly lowered in multiple sclerosis patients as well [83]. In the absence of Tec family kinase ITK, a CD28 downstream signaling molecule, there was a profound diminishment of islet-infiltrating inflammatory cells in mice with T1DM [84].

Taken together, B7/CD28 co-stimulation has divergent effects on the pathogenesis of diabetes mellitus in the different context of disease, which leads to a great barrier for the therapeutic method in diabetes. While not only complicated role of B7/CD28 dyad, many other co-stimulatory molecule dyads also exhibit a dichotomous role in the pathogenesis of diabetes. Below, we discuss some other co-stimulatory molecules that play an essential role in diabetes development.

### Complicated role of other co-stimulatory molecules in diabetes

#### ICOS

Inducible co-stimulator (ICOS), a member of the CD28 family, is expressed after T cell activation [85]. The deletion of ICOS in T cells results in a decreased production of the Th1 cytokine IFN-γ without affecting the numbers of regulatory T cells. ICOS plays a considerable role in the induction of the autoimmune-mediated diabetes [86]. However, there was also a study reporting that the absence of ICOS exacerbates the disease activity in experimental models of diabetes by ablating Treg function [87]. This difference might be caused by the different function of ICOS on different cells, which leads to discrepant outcome.

#### B7-H4

A member of the B7 family, is expressed on the cell membrane of APCs and up-regulated when they activated by exogenous and endogenous stimulator [88, 89]. However, its specific receptors remain unknown. Previous study showed that B7-H4 deficiency increased the incidence and severity of EAE and collagen-induced arthritis (CIA) [90–92]. Furthermore, B7-H4 inhibits islet allograft rejection and decreases lymphocyte proliferation [93]. Recent studies also indicate a suppressive function of B7-H4 in the development of diabetogenic autoimmunity. An increased level of soluble B7-H4 (sVTCN1) was detected in T1DM patients, which is correlated with the aggressive pace of disease. The sVTCN1 lost its immunosuppressive function on inhibiting diabetogenic T cells. Therefore, inhibiting the cleavage of membrane B7-H4 may serve as a potential therapeutic strategy [94, 95]. Independent of inhibiting the recruitment of activated CD4 + and CD8 + T cells to islets, B7-H4 Ig treatment significantly postponed the disease onset and reduced incidence of diabetes in NOD mice due to a transient increase of Treg cells population [96]. Furthermore, β cell-specific B7-H4 overexpression protected against allograft rejection [97]. Unexpectedly, endogenous B7-H4 showed a defect in inhibitory costimulation, but augments the activation of diabetogenic T cell during T1D development [95]. Further study should be carried out to address the exact role of B7-H4 in the immune modulation during the development of diabetes.

#### CD40/CD40L

The costimulatory molecule CD40 and its ligand CD40L (CD154) are expressed by T cells, B cells, APCs, pancreatic islet β cells, and pancreatic ductal cells [12, 98]. In T1DM animal model NOD mice, blockage of CD40L during early diabetes ameliorates spontaneous disease onset, resulting from the decreased number of auto-reactive T cells [99–101]. In parallel with T1DM, CD40-CD40L interactions showed a pro-inflammatory role of in adipose tissue inflammation. Deletion of CD40L protected against weight gain, adipose tissue inflammation, hepatosteatosis, and insulin resistance after high-fat diet feeding [102–105]. However, it has been demonstrated that CD40-/- mice on high-fat diet displayed increased weight gain, impaired insulin secretion, and upregulated pro-inflammatory cytokines compared to the wild type mice. Further data revealed that the expression of pro-inflammatory cytokines inhibited by CD40 activation only found in T cells, but not in B cells or macrophages. This study provided the evidence that protective effect of CD40 was closely associated with CD40 signaling on T cells, which improved adipose tissue inflammation and metabolic complications [106]. These data suggest CD40L/CD40 also plays a complicated role in the development of obesity.

#### 4-1BB/4-1BBL

As a member of the TNF receptor superfamily, 4-1BB provides a co-stimulatory signal through binding to its ligand 4-1BBL [12, 107]. 4-1BB is expressed on adipocytes and macrophages, and is upregulated by obesity-related factors [108]. 4-1BB and/or 4-1BBL agonists activate inflammatory signaling molecules in adipocytes and macrophages [109]. In consistency, 4-1BB deficiency protects against HFD-induced obesity, glucose intolerance, and fatty liver disease though decrease adipose infiltration of macrophages/T cells, and tissue levels of inflammatory cytokines [110]. Unexpectedly, anti-4-1BB scFv transgenic NOD mice developed more severe diabetes than their non-transgenic littermates, as evidenced by earlier onset, faster diabetic process, and higher mortality rate [111].

## CONCLUSIONS

Heretofore, although lots of basic and clinical studies of co-stimulatory molecules have been investigated in the pathogenesis of diabetes, the roles and mechanisms remains ill defined. Due to the complicated and dedicated micro-environment of disease, contradictory role of co-stimulatory dyads is often observed in the development of diabetes. The possible reasons for the contradictory roles of co-stimulatory dyads in diabetes mellitus might be as follow: 1) The basal expression of co-stimulatory molecules such as B7-1 and B7-2 is required to prevent heightened inflammatory response by sustaining Treg populations; 2) The expression of different co-stimulatory molecules may be regulated differentially by a variety of inflammatory cytokines during the process of diabetes; 3) Different co-stimulatory molecules have distinct effects on different cell populations, which leads to discrepant outcomes; 4) The source (exogenous versus endogenous) of co-stimulatory molecules such as B7-H4 might affect their functions on immune activation; 5) In addition, the intervention methods to block co-stimulatory molecules (eg. Antibody-mediated neutralization and administration of recombinant fusion proteins) might affect the function of other co-stimulatory molecules. For example, CTLA-4 depletion also promotes the ligation between B7 and CD28. Therefore, further studies are required to fully understand the pathophysiological roles of co-stimulation in diabetes and develop immunomodulatory therapeutics against the inflammatory process in metabolic disease.
